# Vancomycin‐Resistant *Enterococcus* in Wild Animals: A Global Scoping Review

**DOI:** 10.1002/mbo3.70292

**Published:** 2026-04-22

**Authors:** Yahye Ahmed Nageye, Abdirasak Sharif Ali Mude, Kizito Eneye Bello

**Affiliations:** ^1^ Faculty of Medicine and Health Sciences SIMAD University Mogadishu Somalia; ^2^ Department of Microbiology, Faculty of Natural Science Kogi State (Prince Abubakar Audu) University, Anyigba Anyigba Kogi State Nigeria

**Keywords:** antimicrobial resistance, one health approach, vancomycin‐resistant enterococcus, wildlife reservoirs

## Abstract

Antimicrobial resistance is a global One Health concern, and vancomycin‐resistant *Enterococcus* (VRE) remains one of the clinically important resistant bacterial groups. Although VRE has been extensively studied in clinical and livestock settings, it has been investigated far less often in free‐ranging wild animals. This scoping review maps the published evidence on VRE in wild animals, with emphasis on reported prevalence, host and environmental correlates, anthropogenic exposures, and laboratory detection methods rather than quantifying pooled effect sizes. A structured search of PubMed, ScienceDirect, Scopus, Web of Science, and Google Scholar was conducted from database inception to the final search stage used for this review, with only peer‐reviewed English‐language primary studies contributing empirical wildlife VRE data included in the synthesis. Eligibility was guided by the Population–Concept–Context framework, and the mapped evidence included mammals plus one reptile study retained in the review scope. Twenty‐five studies met the inclusion criteria. Most were conducted in Europe, with substantially fewer studies from Africa and South America, indicating major geographical gaps. Reported prevalence ranged from 1.08% in feral pigs in Brazil to 100% in wild rabbits in Spain, although several striking estimates were based on very small samples and should be interpreted cautiously. Culture‐based methods were most frequently used, whereas PCR‐based approaches were particularly useful for confirming resistance genes. Overall, the evidence suggests that wild animals can act as reservoirs or sentinels of VRE exposure in anthropogenically influenced environments. Standardized surveillance, clearer reporting, and broader One Health monitoring that explicitly includes wildlife are needed.

## Graphical abstract

Vancomycin‐resistant enterococci in free‐ranging wild animals: A systematic scoping review.

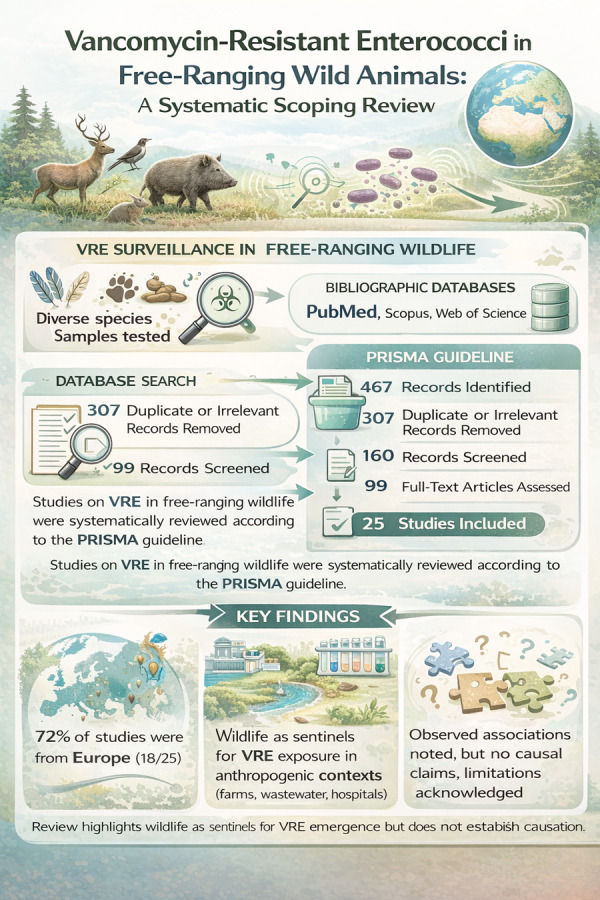

## 1 Introduction

Antimicrobial resistance (AMR) is a major public‐health and One Health challenge because resistant organisms and resistance genes circulate across humans, animals, food systems, and environmental compartments (Lozano, González‐Barrio, García, et al. 2015; Manson et al. 2003; Phukan et al. 2016; Miller et al. 2020; Das et al. 2022; Lawpidet et al. 2021). *Enterococcus faecalis* and *Enterococcus faecium* are particularly relevant because they combine ecological hardiness with the capacity to acquire and exchange resistance determinants. Vancomycin‐resistant *Enterococcus* (VRE) is therefore important not only in hospitals, where it limits treatment options, but also in extra‐clinical reservoirs that may sustain persistence and dissemination. A scoping review is appropriate for this topic because the wildlife VRE literature is heterogeneous in host taxa, settings, and laboratory methods, and is not yet suited to a narrow effect‐estimation question. The purpose is to map the breadth and characteristics of the evidence base, identify knowledge gaps, and summarize reported correlates rather than test a single causal hypothesis (Miller et al. 2020; Das et al. 2022; Lawpidet et al. 2021). Enterococci are common commensals of the intestinal tract of humans and animals, yet they can also behave as opportunistic pathogens. Their intrinsic resilience, environmental persistence, and ability to acquire van genes make them informative indicators of AMR movement across ecological interfaces (Miller et al. 2020; Das et al. 2022; Lawpidet et al. 2021). For this reason, wildlife surveillance is relevant even when the immediate public‐health risk cannot be quantified directly from wildlife prevalence data alone (Lozano et al. 2016a; Azzam et al. 2023; Smout et al. 2023). Most VRE research has focused on healthcare, livestock, food, and wastewater systems, while wildlife remains comparatively understudied (Lozano et al. 2016a; Azzam et al. 2023; Smout et al. 2023; Radhouani et al. 2011a). Nonetheless, free‐ranging wildlife may be exposed to resistant enterococci through contaminated water, agricultural runoff, refuse, carcasses, and contact zones linking wild fauna, domestic animals, and human settlements. Wildlife may therefore function as reservoirs, spillover recipients, or sentinels of anthropogenic AMR contamination within a One Health framework (Radhouani et al. 2011a; Chhatwal et al. 2020). The mechanisms linking wildlife and VRE are biologically plausible but are not uniformly measured in the included studies. Across the broader AMR literature, wastewater discharge, manure application, environmental contamination, and access to human‐associated food sources are frequently discussed as potential pathways of exposure (Radhouani et al. 2011a; Chhatwal et al. 2020; Meena et al. 2017; Raza et al. 2018; Liu et al. 2020; Chen et al. 2019). In the present review, these pathways are treated primarily as reported correlates or hypothesized drivers unless they were directly measured within an included wildlife study. The available wildlife literature also appears geographically uneven. Published studies are concentrated in Europe, with relatively limited evidence from Africa, South America, and Asia, making it difficult to determine whether observed regional differences reflect true epidemiological variation or uneven surveillance intensity (Meena et al. 2017; Raza et al. 2018; Liu et al. 2020). This gap is important because wildlife–livestock–environment interfaces differ markedly across settings (Chen et al. 2019). Host ecology may also shape observed VRE occurrence. Omnivorous, scavenging, peri‐urban, and water‐associated species are often discussed as more exposed because of their feeding habits and habitat use, but evidence remains mainly descriptive. Apparent high prevalence in some species should be interpreted in light of sample size and study design rather than assumed to represent stable ecological niches (Kampmeier et al. 2020). Another challenge is methodological heterogeneity. Studies differ in host species, sample matrices, susceptibility methods, resistance confirmation, and reporting practices, which limits direct comparison. Accordingly, a scoping review is appropriate because it maps what has been studied, where, and how, while identifying evidence gaps rather than estimating pooled effect sizes.

This revised review therefore aligns its scope explicitly with wild animals rather than wild mammals alone, acknowledges that the evidence base includes one reptile study, and interprets reported patterns cautiously. The aim is to summarize the existing evidence, clarify what is directly supported by the included wildlife studies, and highlight priorities for surveillance and future research.

### 1.1 Objectives of This Scoping Review

This scoping review aimed to:

1. map the published evidence on VRE occurrence in free‐ranging wildlife globally;
2. summarize reported host, environmental, and anthropogenic correlates and hypothesized drivers discussed in the included studies;
3. describe the laboratory and surveillance methods used to detect VRE in wildlife and identify areas of methodological inconsistency;
4. summarize how study quality was appraised and how those appraisals informed interpretation, without excluding studies on the basis of quality;
5. identify geographical, taxonomic, and methodological gaps relevant to One Health surveillance and future research.

### 1.2 Scoping Review Research Questions

1. What evidence has been published on VRE occurrence in free‐ranging wildlife, and which regions and host taxa have been studied?
2. Which correlates and hypothesized drivers of VRE occurrence are reported in the included studies, particularly in relation to anthropogenic pressures and wildlife ecology?
3. Which sampling, culture‐based, and molecular methods have been used to identify VRE in wildlife, and what methodological gaps limit comparability across studies?

## 2 Methods

### 2.1 Search Strategy and Databases

Searches were conducted in PubMed, ScienceDirect, Scopus, Web of Science, and Google Scholar using combinations of terms related to “vancomycin‐resistant *Enterococcus*,” wildlife, wild animals, wild mammals, prevalence, AMR, and reservoir hosts. No publication‐year limits were applied; accordingly, the search covered records from database inception to the final search stage used for this review. Because Google Scholar returns a very large volume of records, screening was limited to the first 200 results ranked by relevance. Only English‐language studies were considered for inclusion. Gray literature was screened only to identify contextual information and potentially relevant citations, but gray‐literature records were not eligible for inclusion in the final evidence map. Details of the search strategy are provided in Supporting Information File K1.

### 2.2 Eligibility Criteria

Eligibility was defined using the Population–Concept–Context (PCC) framework. In this revised manuscript, the scope is stated consistently as wildlife, because the included evidence base contains studies on free‐ranging mammals and one reptile (Russian tortoise).

Population (P): Free‐ranging wildlife species. Studies focused exclusively on humans, livestock, pets, captive zoo collections, or agricultural animals were excluded unless wildlife‐specific data were separately extractable.

Concept (C): Occurrence of VRE in wildlife, including prevalence or detection data, laboratory detection methods, resistance characteristics, and author‐reported ecological or anthropogenic correlates. Studies without empirical VRE data were excluded.

Context (C): Wildlife sampled in natural, peri‐urban, aquatic, terrestrial, or other human‐influenced environments worldwide. Clinical‐only, hospital‐only, and agriculture‐only studies without a wildlife component were excluded.

Included records were peer‐reviewed primary studies published in English. Excluded records comprised reviews, editorials, conference abstracts, case reports, and gray‐literature items from the mapped synthesis. No studies were excluded on the basis of quality appraisal scores. Because no prospectively registered or publicly available review protocol was available, this is acknowledged as a limitation of the review process.

### 2.3 Study Selection Process

After duplicate removal (*n* = 2186), 911 records were screened by title and abstract. Of 128 full‐text reports assessed for eligibility, 25 met the inclusion criteria. Screening was conducted by the review team using the predefined eligibility criteria, and uncertainties were resolved through discussion among the authors. The principal reasons for full‐text exclusion were the absence of explicit VRE data (*n* = 29), unclear wildlife source or origin of isolates (*n* = 65), and noneligible publication type (*n* = 9). The PRISMA‐style flow diagram was reviewed for internal consistency and is presented as a concise summary of the selection process.

### 2.4 Data Extraction

Data were extracted into a standardized form, including author and year, country, wildlife species, sample type, sample size, number positive, reported prevalence, detection method, and a flag indicating estimates based on small samples. The extraction form was piloted on a subset of included studies and then applied across the full data set, with extracted entries cross‐checked for completeness and consistency. Additional notes were recorded on the ecological context and any author‐reported correlates or hypothesized drivers.

### 2.5 Data Synthesis

Descriptive synthesis was used to summarize patterns by region, host species, and laboratory method. This scoping review maps the available evidence rather than quantifying pooled effect sizes. Critical appraisal skills programme (CASP) and mixed methods appraisal tool (MMAT) tools were used only to describe study quality and inform the cautious interpretation of findings; they were not used to exclude or statistically weight studies. The absence of meta‐analysis was a methodological choice consistent with the scoping objective and marked heterogeneity of the evidence base, rather than a limitation in itself.

## 3 Results

### 3.1 Overview of Included Studies

**Figure 1:**
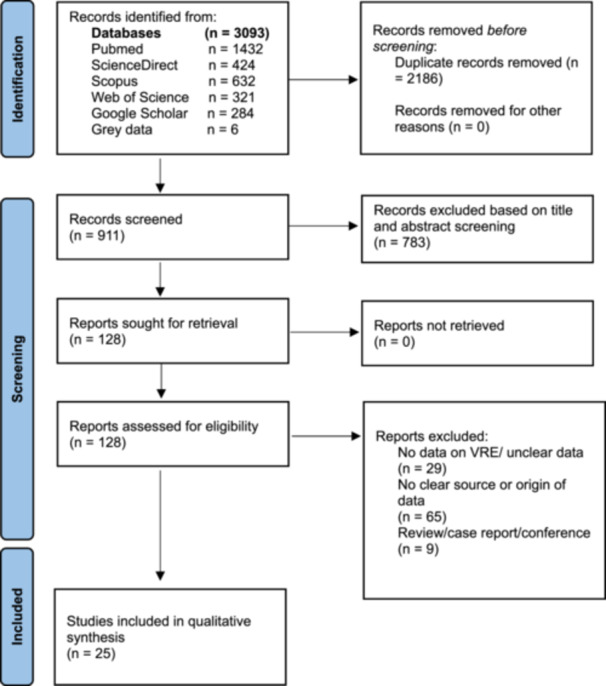
Summary of the studies selection and screening process. VRE, vancomycin‐resistant *Enterococcus*.

The review included 25 studies reporting VRE in free‐ranging wildlife from Europe, Africa, and South America (Figure 1). Europe contributed the majority of studies, whereas Africa and South America were sparsely represented, and little to no evidence was identified from large parts of Asia. The evidence base was dominated by cross‐sectional designs and descriptive prevalence studies; few studies were designed to test risk factors quantitatively.

Sample sizes ranged from 2 to 283 animals (Table 1). Most records concerned mammals, but the data set also contained one reptile study (Russian tortoise), which is why the scope of this revised manuscript is framed consistently as wildlife rather than mammals alone. Extreme prevalence values should be interpreted cautiously when derived from very small samples.

**Table 1:** Characteristics of the included studies, with a small‐sample caution flag.

S/N	Author, publication year	Country	Wildlife type	Sample type	Sample size	Number positive	Prevalence (%)	Detection method
1	Mallon et al. (2002)	England	Wood mice	Fecal	120	6	5.00	Broth microdilution
2	Mallon et al. (2002)	England	Badger	Fecal	124	2	1.61	Enterococcosel agar
3	Poeta et al. (2005)	Portugal	Mouflon	Fecal	140	4	2.86	PCR
4	Poeta et al. (2005)	Portugal	Fox	Fecal	140	2	1.43	PCR
5	Poeta et al. (2007)	Portugal	Wild boars	Fecal	67	2	2.99	Agar dilution
6	Poeta et al. (2008)	Portugal	Wild mammals	Fecal	134	2	1.49	PCR
7	Figueiredo et al. (2009)	Portugal	Wild rabbits	Fecal	77	11	14.29	Agar dilution
8	Lessa et al. (2011)	Brazil	Feral pigs	Fecal	186	2	1.08	Disk diffusion
9	Radhouani et al. (2011b)	Portugal	Red foxes	Fecal	52	7	13.46	Agar dilution
10	Semedo‐Lemsaddek et al. (2013)	Portugal	Eurasian otters	Fecal	29	18	62.07	Disk diffusion
11	Katakweba et al. (2015)	Tanzania	Buffalo	Fecal	35	1	2.86	Disk diffusion
12	Katakweba et al. (2015)	Tanzania	Zebra	Fecal	40	8	20.00	Disk diffusion
13	Katakweba et al. (2015)	Tanzania	Wildebeest	Fecal	40	1	2.50	Disk diffusion
14	Nowakiewicz et al. (2014)	Poland	Wild mammals	Swabs	70	7	10.00	Agar dilution
15	Nowakiewicz et al. (2014)	Poland	Russian tortoise	Swabs	17*	6	35.29	Agar dilution
16	Lozano, Gonzalez‐Barrio, Camacho, et al. (2015)	Spain	Black rats	Fecal	46	8	17.39	PCR
17	Lozano, Gonzalez‐Barrio, Camacho, et al. (2015)	Spain	Wood mice	Fecal	41	2	4.87	PCR
18	Lozano, Gonzalez‐Barrio, Camacho, et al. (2015)	Spain	Common voles	Fecal	54	1	1.85	PCR
19	Lozano, Gonzalez‐Barrio, Camacho, et al. (2016)	Spain	Wild boars	Swabs	81	5	6.17	PCR
20	Guerrero‐Ramos et al. (2016)	Spain	Roe deer	Meat	35	17	48.57	Disk diffusion
21	Guerrero‐Ramos et al. (2016)	Spain	Wild boars	Meat	14*	7	50.00	Disk diffusion
22	Guerrero‐Ramos et al. (2016)	Spain	Wild rabbits	Meat	2*	2	100.00	Disk diffusion
23	Dec et al. (2020)	Italy	Wolf	Fecal	52	3	5.77	Broth microdilution
24	Hamarova et al. (2021)	Slovakia	Wild mammals	Fecal	283	16	5.78	Disk diffusion
25	Smoglica et al. (2023)	Italy	Apennine chamois	Fecal	48	1	2.08	VITEK 2

Abbreviation: PCR, polymerase chain reaction.

* Sample sizes marked with an asterisk were based on *n* < 20 and should be interpreted cautiously.

### 3.2 VRE Prevalence in Wildlife

**Figure 2:**
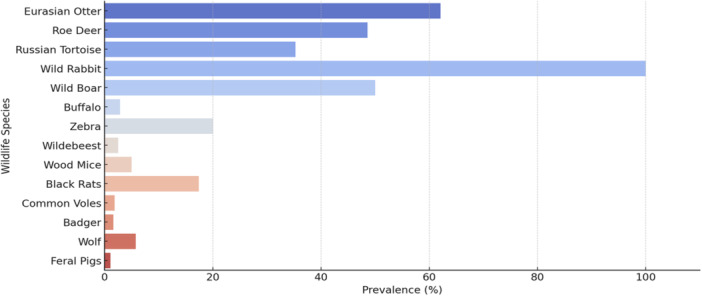
Prevalence of vancomycin‐resistant *Enterococcus* among wildlife species.

Reported VRE prevalence varied widely across studies, from 1.08% in feral pigs in Brazil to 100% in wild rabbits in Spain (Figure 2). However, some of the most striking estimates were derived from very small samples and should not be interpreted as robust indicators of stable high‐prevalence niches. In particular, the 100% estimate for wild rabbits was based on *n* = 2, and the 35.29% estimate for Russian tortoises was based on *n* = 17.

#### 3.2.1 Species Patterns

Higher reported prevalence was observed in Eurasian otters (62.07%; *n* = 29), roe deer (48.57%; *n* = 35), and Russian tortoises (35.29%; *n* = 17), but these figures should be interpreted in relation to sample size, study setting, and method. The evidence is more consistent with possible species‐ and habitat‐linked exposure differences than with definitive species‐level risk ranking. The wild‐rabbit estimate of 100% is especially uncertain because it was based on only two samples.

#### 3.2.2 Geographic Trends

**Figure 3:**
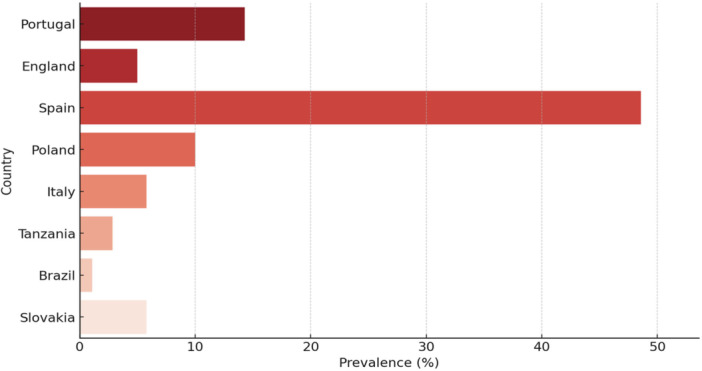
Prevalence of vancomycin‐resistant *Enterococcus* in relation to country.

European studies reported a broader range of VRE prevalence than studies from Africa and South America (Figure 3). These patterns may reflect differences in surveillance intensity, host assemblages, agricultural practices, environmental contamination, and laboratory methods; they should not be interpreted as causal or as evidence that Europe necessarily has higher wildlife VRE burdens. The currently mapped literature is too sparse and heterogeneous for that inference.

#### 3.2.3 Detection Methods

**Figure 4:**
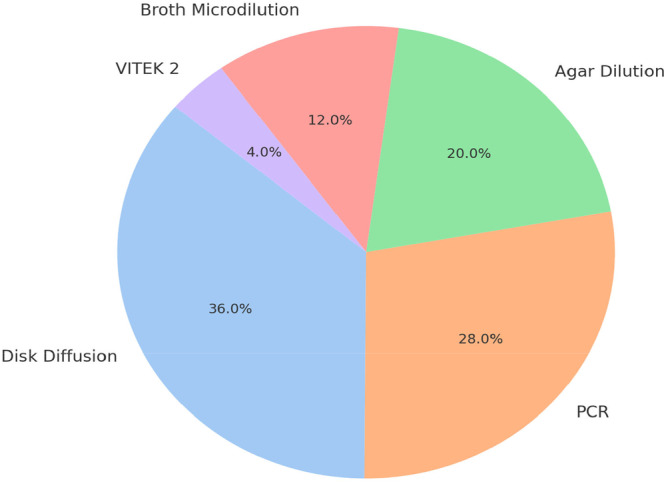
Prevalence of vancomycin‐resistant *Enterococcus* in relation to method of detection. PCR, polymerase chain reaction.

The included studies used a mix of culture‐based and molecular methods, including disk diffusion, agar dilution, broth microdilution, polymerase chain reaction (PCR), and, in one study, VITEK 2 (Figure 4). Because methods differed in sensitivity, specificity, and reporting detail, prevalence estimates should be compared cautiously across studies. In this review, method differences are described as part of the mapped evidence rather than treated as directly comparable performance estimates.

Culture‐based approaches were most frequently used, whereas PCR‐based methods were particularly useful for confirming resistance genes. The heterogeneity of methods reinforces the need for more standardized wildlife AMR surveillance protocols and clearer reporting of resistance‐confirmation pathways. As a minimum core protocol for future studies, wildlife VRE surveys should clearly report sampling matrix, selective culture conditions, susceptibility testing method, interpretive standard, and molecular confirmation of key van genes where feasible.

### 3.3 Reported Correlates and Hypothesized Drivers of VRE Occurrence in Wildlife

**Figure 5:**
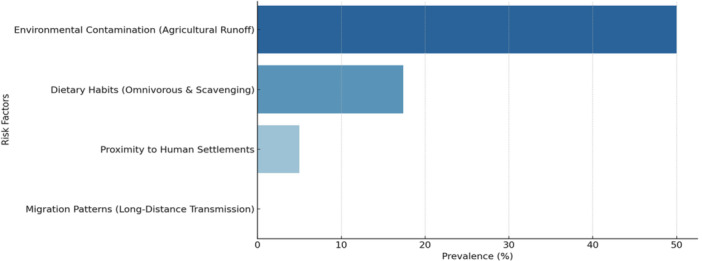
Prevalence of vancomycin‐resistant *Enterococcus* in relation to risk factors.

The included studies and their ecological discussion most often linked wildlife VRE occurrence with environmental contamination, proximity to human settlement, feeding behavior, and exposure to agricultural landscapes (Figure 5). Because these variables were usually inferred from study context rather than measured analytically, they are interpreted here as reported correlates or hypothesized drivers rather than quantified risk factors.

Omnivorous, scavenging, peri‐urban, and water‐associated species were repeatedly discussed as potentially more exposed. These patterns are consistent with broader AMR literature, but the included wildlife VRE studies were predominantly descriptive and generally not designed to estimate effect sizes for such correlates.

### 3.4 Anthropogenic Activities and VRE Occurrence

Across the included studies, author discussions frequently associated wildlife VRE occurrence with anthropogenic pressures, such as agricultural runoff, wastewater discharge, urbanization, and inadequate waste management. The evidence suggests these factors may be associated with wildlife exposure, but the cross‐sectional nature of most studies means that causal attribution remains limited.

Climate, habitat fragmentation, and other environmental processes may also influence bacterial persistence and contact structure, but direct evidence from the 25 included wildlife VRE studies is limited. Accordingly, these mechanisms are presented as plausible pathways drawn partly from the broader AMR literature and not as findings that were consistently tested within the included wildlife studies.

**Figure 6:**
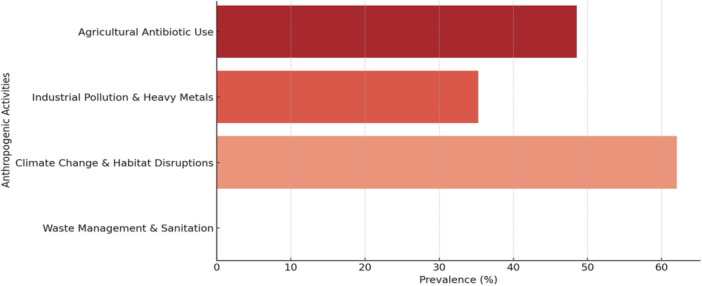
Prevalence of vancomycin‐resistant *Enterococcus* among wildlife species in relation to anthropogenic activities.

Figure 6 should therefore be interpreted as a descriptive visualization of author‐reported anthropogenic contexts rather than a quantitative ranking of causal importance. Agricultural exposure was the most frequently discussed context, but this reflects the narrative emphasis of the included literature as much as formal comparative testing.

Semiaquatic and peri‐urban species, such as Eurasian otters, foxes, and rodents, were often highlighted in relation to polluted waters or waste‐associated exposure pathways. These patterns are suggestive and useful for surveillance planning, but they do not establish causation.

### 3.5 Heatmaps and Figure Interpretation

**Figure 7:**
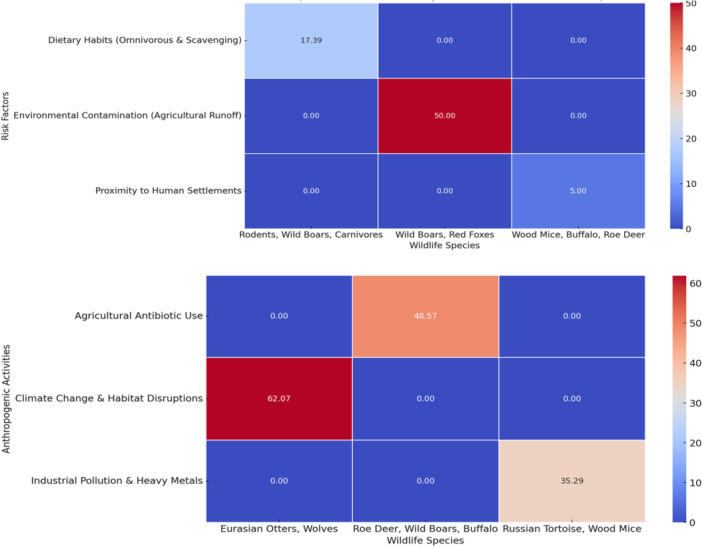
Heatmap of vancomycin‐resistant *Enterococcus* prevalence in relation to risk factors and Anthropogenic Activities.

Figures 5, 6, 7 are intended as descriptive summaries to help visualize recurring patterns across studies. They should not be read as implying precise quantitative comparability across species, countries, or exposure categories because the underlying data are heterogeneous and often based on small samples.

Two broad patterns are more defensible than detailed bar‐by‐bar interpretation: first, many studies discussed anthropogenic exposure pathways around agriculture, waste, and polluted water; second, several omnivorous, scavenging, peri‐urban, or water‐associated wildlife taxa appeared repeatedly in those contexts. Stronger inference would require more standardized and analytically designed studies.

### 3.6 Relation of Wildlife Occurrence to Anthropogenic Activities

The heatmap relating wildlife occurrence to anthropogenic activities again indicates that agricultural landscapes, waste exposure, and disturbed habitats were recurrently discussed across the studies. These are best interpreted as mapped contextual themes rather than demonstrated species‐specific risk factors.

Overall, the visualization supports the conclusion that wildlife AMR surveillance should prioritize human‐influenced interfaces, especially aquatic systems, peri‐urban habitats, and mixed wildlife‐livestock landscapes, while avoiding overinterpretation of single‐study or small‐sample estimates.

## 4 Thematic Interpretation

The themes identified across studies are summarized here to support interpretation rather than to restate the results. In keeping with the scoping purpose, these themes are framed as descriptive syntheses of recurring patterns and hypothesis‐generating interpretations, not as quantified causal effects. This interpretive section is kept brief to avoid duplication with the Results and Discussion.

### 4.1 Anthropogenic and Environmental Contexts

Many included studies discussed agricultural runoff, manure use, wastewater discharge, and polluted water bodies as plausible wildlife exposure pathways. These patterns are consistent with the broader AMR literature, but only a minority of the wildlife VRE studies directly measured these pathways. The evidence, therefore, supports cautious statements such as “may be associated with” or “is consistent with” rather than strong causal claims.

### 4.2 Urbanization, Habitat Overlap, and Food‐Associated Exposure

Peri‐urban wildlife, scavengers, and omnivores were repeatedly described as likely to encounter resistant bacteria via refuse, carcasses, contaminated water, or anthropogenic food sources. Such ecological interpretations are biologically plausible and useful for hypothesis generation, yet they remain largely descriptive within the included evidence base.

### 4.3 Cross‐Species Interfaces and One Health Relevance

The mapped literature supports the relevance of wildlife to One Health AMR surveillance because wildlife can reflect contamination at interfaces linking humans, livestock, water systems, and the broader environment. However, the included studies generally document occurrence rather than transmission direction, so wildlife should be framed as potential reservoirs, sentinels, or interface hosts rather than assumed sources of onward spread.

### 4.4 Methodological Implications

A recurring theme was the lack of standardized sampling and resistance‐confirmation methods. Variation in specimen type, susceptibility testing, confirmation of van genes, and reporting structure limits comparability and makes pooled interpretation difficult. Longitudinal and analytically designed studies are needed to move beyond descriptive mapping toward stronger inference about determinants of wildlife VRE occurrence.

### 4.5 Quality Appraisal and How It Informed Interpretation

CASP and MMAT assessments were included to describe the methodological profile of the literature, not to exclude or weight studies. No study was removed on the basis of quality appraisal. In practical terms, CASP scores of 7–9/10 indicate that a study addressed most core appraisal domains but may still have limits in sampling, measurement, or reporting, whereas MMAT scores of 3–5/5 indicate moderate to stronger methodological completeness rather than freedom from bias. Lower‐scoring studies, studies with very small samples, and geographically narrow studies were interpreted more cautiously in the narrative synthesis.

The appraisal findings informed how strongly results were interpreted. In particular, lower‐scoring studies, geographically narrow studies, and studies with very small samples were treated cautiously and were not used to support strong claims about prevalence hotspots, species‐specific risk, or causal drivers. This approach is consistent with the purpose of a scoping review, which maps evidence while acknowledging its limitations.

Overall, the included literature provides useful signals for surveillance and hypothesis generation, but the strength of inference remains constrained by cross‐sectional designs, heterogeneous methods, and uneven geographic coverage.

## 5 Discussion

This scoping review mapped 25 wildlife studies reporting VRE and showed that the current evidence base is taxonomically and geographically uneven. Most studies originated from Europe, while Africa and South America were represented by only a few studies and evidence from much of Asia was absent. This imbalance limits broad geographic comparison and highlights important surveillance gaps within a One Health framework. The review also showed that apparent differences across host species must be interpreted cautiously. Although higher prevalence was reported in some species and settings, the evidence is dominated by cross‐sectional studies and several notable estimates were based on small samples. Accordingly, the findings are better interpreted as signals of potentially important interfaces or exposure contexts than as definitive rankings of species‐level risk.

Reported associations with agricultural landscapes, wastewater, waste exposure, urbanization, and aquatic habitats were recurrent across the literature, but these were usually discussed as ecological context rather than tested analytically. For that reason, the manuscript now refers to correlates and hypothesized drivers instead of broad “risk factors” unless a study directly measured an exposure‐response relationship. The included studies nevertheless support the idea that wildlife can participate in AMR ecology as interface hosts or sentinels of anthropogenic contamination. Semiaquatic species, scavengers, omnivores, and peri‐urban wildlife appear especially relevant for surveillance planning because they repeatedly occur at contact points linking wildlife, humans, livestock, and contaminated environments. Methodological heterogeneity remained a major barrier to synthesis. Differences in sample type, sample size, resistance testing, and gene confirmation complicate comparison across studies and reduce confidence in apparent prevalence contrasts. Standardized protocols for sampling, susceptibility testing, molecular confirmation, and reporting would substantially improve the interpretability of wildlife VRE surveillance. In this context, the lack of meta‐analysis should be seen as a deliberate methodological choice aligned with the scoping design rather than as a weakness of the review.

A further implication is that figures and summary tables should be read as descriptive tools rather than precise quantitative summaries. The present review, therefore, emphasizes only broad patterns that are reasonably supported by the mapped evidence and avoids strong causal wording where direct analytic evidence was lacking.

The literature supports inclusion of wildlife in AMR monitoring systems, but it does not yet permit robust quantification of determinants, transmission direction, or comparative regional burden. Future work should prioritize longitudinal, multiregional, and analytically designed studies, especially in underrepresented regions, to clarify how wildlife fits within wider VRE ecology.

This interpretation strengthens the One Health relevance of the review while keeping the conclusions proportional to the underlying evidence base.

### 5.1 Strengths and Limitations

Strengths of this review include its explicit scoping approach, global perspective, inclusion of study‐quality appraisal, and revised effort to distinguish directly reported findings from broader ecological interpretation. The review also makes visible important evidence gaps across regions, host taxa, and laboratory approaches.

The main limitations are the heterogeneous and predominantly cross‐sectional nature of the underlying studies, inconsistent laboratory and reporting methods, possible publication and language bias from the English‐only inclusion criterion, the exclusion of gray literature from the mapped synthesis, and the absence of a prospectively registered review protocol. In addition, some prevalence estimates were based on very small samples, which limits the robustness of species‐level inferences.

## 6 Conclusion

This scoping review maps the available evidence on VRE in free‐ranging wild animals and shows that the literature is concentrated in Europe, with notable gaps in Africa, South America, and much of Asia. The mapped studies suggest that wild animals may act as reservoirs, interface hosts, or sentinels of VRE exposure in human‐influenced environments, but the current evidence is mainly descriptive and does not quantify effect sizes or establish causation. The review adds value by clarifying scope, highlighting major geographical and methodological gaps, emphasizing cautious interpretation of small‐sample estimates, and identifying the need for standardized methods and integrated wildlife surveillance within One Health AMR frameworks. Progress will depend on better geographic coverage, longitudinal study designs, harmonized laboratory workflows, and clearer reporting of ecological context and exposure pathways.

## Author Contributions

**Yahye Ahmed Nageye:** conceptualization, methodology, visualization, supervision, writing and editing of the manuscript. **Abdirasak Sharif Ali:** conceptualization, investigation, funding acquisition, writing – original draft, methodology. **Kizito Eneye Bello:** validation, visualization, supervision.

## Funding

The authors have nothing to report.

## Ethics Statement

The authors have nothing to report.

## Consent

The authors have nothing to report.

## Conflicts of Interest

The authors declare no conflicts of interest.

## Supporting information

Supporting File

## Data Availability

The data presented in this study are available in the supplementary material.
