# Supplementary material for: Vancomycin‐Resistant Enterococcus in Wild Animals: A Global Scoping Review
Source: Microbiologyopen. 2026 Apr 22;15(2):e70292. doi: 10.1002/mbo3.70292 (PMC13102680; doi:10.1002/mbo3.70292)
Supplement: Supplementary file 1 — Supporting File [file MBO3-15-e70292-s001.pdf]

FILE K1: SEARCH STRATEGY

PubMed

((((((((((((Vancomycin resistant enterococcus)) OR (VRE)) OR (Vancomycin resistant enterococci)) OR (antibiotic resistance)) AND (virulence traits)) AND (resistant genes)) AND (wild mammals)) AND (wild animals)) AND (mammals)) AND (antimicrobial resistance)) AND (prevalence)) OR (epidemiology)) AND (AMR)

((((((((((((Vancomycin resistant enterococcus)) OR (VRE)) OR (Vancomycin resistant enterococci)) OR (antibiotic resistance)) AND (virulence traits)) AND (resistant genes)) AND (wild mammals)) AND (wild animals)) AND (mammals)) AND (antimicrobial resistance)) AND (prevalence)) OR (epidemiology)) AND (AMR)

MESH TERMS

((("vancomycin resistant enterococci"[MeSH Terms] OR ("vancomycin resistant"[All Fields] AND "enterococci"[All Fields]) OR "vancomycin resistant enterococci"[All Fields] OR ("vancomycin"[All Fields] AND "resistant"[All Fields] AND "enterococcus"[All Fields]) OR "vancomycin resistant enterococcus"[All Fields] OR "VRE"[All Fields] OR ("vancomycin resistant enterococci"[MeSH Terms] OR ("vancomycin resistant"[All Fields] AND "enterococci"[All Fields]) OR "vancomycin resistant enterococci"[All Fields] OR ("vancomycin"[All Fields] AND "resistant"[All Fields] AND "enterococci"[All Fields]) OR "vancomycin resistant enterococci"[All Fields]) OR ("drug resistance, microbial"[MeSH Terms] OR ("drug"[All Fields] AND "resistance"[All Fields] AND "microbial"[All Fields]) OR "microbial drug resistance"[All Fields] OR ("antibiotic"[All Fields] AND "resistance"[All Fields]) OR "antibiotic resistance"[All Fields])) AND (("pathogenicity"[MeSH Subheading] OR "pathogenicity"[All Fields] OR "virulence"[All Fields] OR "virulence"[MeSH Terms] OR "virulences"[All Fields] OR "virulent"[All Fields]) AND ("trait"[All Fields] OR "trait s"[All Fields] OR "traits"[All Fields])) AND (("resist"[All Fields] OR "resistance"[All Fields] OR "resistances"[All Fields] OR "resistant"[All Fields] OR "resistants"[All Fields] OR "resisted"[All Fields] OR "resistence"[All Fields] OR "resistences"[All Fields] OR "resistent"[All Fields] OR "resistibility"[All Fields] OR "resisting"[All Fields] OR "resistive"[All Fields] OR "resistively"[All Fields] OR "resistivities"[All Fields] OR "resistivity"[All Fields] OR "resists"[All Fields]) AND ("gene s"[All Fields] OR "genes"[MeSH Terms] OR "genes"[All Fields])) AND ("wild"[All Fields] AND ("mammal s"[All Fields] OR "mammals"[MeSH Terms] OR "mammals"[All Fields] OR "mammal"[All Fields])) AND ("animals, wild"[MeSH Terms] OR ("animals"[All Fields] AND "wild"[All Fields]) OR "wild animals"[All Fields] OR ("wild"[All Fields] AND "animals"[All Fields])) AND ("mammal s"[All Fields] OR "mammals"[MeSH Terms] OR "mammals"[All Fields] OR

```
"mammal"[All Fields]) AND ("drug resistance, microbial"[MeSH Terms] OR ("drug"[All Fields] AND "resistance"[All Fields] AND "microbial"[All Fields]) OR "microbial drug resistance"[All Fields] OR ("antimicrobial"[All Fields] AND "resistance"[All Fields]) OR "antimicrobial resistance"[All Fields]) AND ("epidemiology"[MeSH Subheading] OR "epidemiology"[All Fields] OR "prevalence"[All Fields] OR "prevalence"[MeSH Terms] OR "prevalance"[All Fields] OR "prevalences"[All Fields] OR "prevalence s"[All Fields] OR "prevalent"[All Fields] OR "prevalently"[All Fields] OR "prevalents"[All Fields])) OR ("epidemiologies"[All Fields] OR "epidemiology"[MeSH Subheading] OR "epidemiology"[All Fields] OR "epidemiology"[MeSH Terms] OR "epidemiology s"[All Fields])) AND ("appl magn reson"[Journal] OR "altern med rev"[Journal] OR "amr"[All Fields])
```

Scopus

TITLE-ABS-KEY(("vancomycin-resistant Enterococcus" OR VRE OR "antimicrobial resistance")
AND ("wild animals" OR wildlife OR "non-domesticated animals")
AND (prevalence OR incidence OR occurrence))

Google scholar
"vancomycin-resistant Enterococcus" AND (wildlife OR "wild animals") AND (prevalence OR incidence)

Science Direct
("vancomycin-resistant Enterococcus" OR VRE OR "antimicrobial resistance")
AND ("wild animals" OR wildlife OR "non-domesticated animals")
AND (prevalence OR incidence OR occurrence)

Web of Science
TS=("vancomycin-resistant Enterococcus" OR VRE OR "antimicrobial resistance")
AND TS=("wild animals" OR wildlife OR "non-domesticated animals")
AND TS=(prevalence OR incidence OR occurrence)

Grey database (WHO)

- "vancomycin-resistant Enterococcus in wildlife"
- "VRE in wild animals global prevalence"
- "Antimicrobial resistance wild animal populations"

File K2: CASP_MMAT_Appraisal SCOPING REVIEW

Study	Study Type	CASP Score (out of 10)	MMAT Score (out of 5)	Key Strengths	Limitations
Guerrero-Ramos et al. (2016)	Observational	8	-	Robust sampling, ecological relevance	Potential bias in observational data
Nowakiewicz et al. (2014)	Quantitative	-	4	Well-defined methodology, reproducible	Limited geographic scope
Poeta et al. (2005)	Qualitative	9	-	Detailed qualitative insights	Small sample size
Hamarova et al. (2021)	Mixed-Methods	-	5	Strong mixed-methods integration	Complexity in mixed-method interpretation
Smoglica et al. (2023)	Quantitative	-	4	Clear statistical analysis	Short study duration
Lozano et al. (2015)	Qualitative	7	-	Rich thematic development	Limited transferability
Semedo-Lemsaddek et al. (2013)	Observational	8	-	Relevant environmental context	Lack of molecular confirmation
Katakweba et al. (2015)	Quantitative	-	3	Good sample size, statistical rigor	Possible data inconsistencies
